# MALDI Imaging Mass Spectrometry of High-Grade Gliomas: A Review of Recent Progress and Future Perspective

**DOI:** 10.3390/cimb45020055

**Published:** 2023-01-18

**Authors:** Alen Rončević, Nenad Koruga, Anamarija Soldo Koruga, Željko Debeljak, Robert Rončević, Tajana Turk, Domagoj Kretić, Tatjana Rotim, Zdravka Krivdić Dupan, Damir Troha, Marija Perić, Tihana Šimundić

**Affiliations:** 1 Department of Neurosurgery, University Hospital Center Osijek, 31000 Osijek, Croatia; 2 Faculty of Medicine, Josip Juraj Strossmayer University of Osijek, 31000 Osijek, Croatia; 3 Department of Neurology, University Hospital Center Osijek, 31000 Osijek, Croatia; 4 Clinical Institute of Laboratory Diagnostics, University Hospital Center Osijek, 31000 Osijek, Croatia; 5 Department of Diagnostic and Interventional Radiology, University Hospital Center Osijek, 31000 Osijek, Croatia; 6 Department of Radiology, Vinkovci General Hospital, 31000 Osijek, Croatia; 7 Department of Clinical Cytology, University Hospital Center Osijek, 31000 Osijek, Croatia; 8 Department of Nephrology, University Hospital Center Osijek, 31000 Osijek, Croatia

**Keywords:** glioblastoma, glioma, mass spectrometry, MALDI-TOF, metabolomics

## Abstract

Glioblastoma (GBM) is the most common malignancy of the brain with a relatively short median survival and high mortality. Advanced age, high socioeconomic status, exposure to ionizing radiation, and other factors have been correlated with an increased incidence of GBM, while female sex hormones, history of allergies, and frequent use of specific drugs might exert protective effects against this disease. However, none of these explain the pathogenesis of GBM. The most recent WHO classification of CNS tumors classifies neoplasms based on their histopathological and molecular characteristics. Modern laboratory techniques, such as matrix-assisted laser desorption/ionization (MALDI) imaging mass spectrometry, enable the comprehensive metabolic analysis of the tissue sample. MALDI imaging is able to characterize the spatial distribution of a wide array of biomolecules in a sample, in combination with histological features, without sacrificing the tissue integrity. In this review, we first provide an overview of GBM epidemiology, risk, and protective factors, as well as the recent WHO classification of CNS tumors. We then provide an overview of mass spectrometry workflow, with a focus on MALDI imaging, and recent advances in cancer research. Finally, we conclude the review with studies of GBM that utilized MALDI imaging and offer our perspective on future research.

## 1. Glioblastoma

### 1.1. Epidemiology

Glioblastoma (GBM), as the most advanced and aggressive form of glial tumors (gliomas), is also the most common malignant tumor of the brain, accounting for 14.5% of all neoplasms of the central nervous system (CNS) and almost half (48.6%) of all malignancies of the CNS [1]. It is also recognized as the highest grade of brain tumor, i.e., grade 4 glioma. Despite many advances in understanding the pathophysiology of high-grade gliomas, improvements in overall survival of patients diagnosed with GBM are almost negligible. The median survival time of these patients from the time of the diagnosis is approximately one year [2], and less than 1% of patients survive for more than 10 years [3]. According to reports, males are affected 1.6-fold more frequently than females and Caucasians are diagnosed with GBM about 2-fold more than African Americans [4]. Despite these discrepancies, there have not been any significant geographical disparities regarding the distribution of newly diagnosed GBM [4]. The median age at diagnosis of GBM increases over time and, lately, is reported to be 64 years [5]; survival rates also decrease with age. The diagnosis of GBM is based on histopathological findings. Furthermore, molecular analyses of GBM are performed in order to better characterize the tumor for prognostic subclassification and individualized treatment options, as recommended by the World Health Organization (WHO) in 2016 [6]. The classification of tumors of the CNS, prior to 2016, was based solely on histological features of the tumor. Although patients were diagnosed with the same type of tumor, outcomes and responses to therapy differed. This suggested submicroscopic distinctions within the same histological class of tumors. In 2016, based on molecular and genetic studies of these neoplasms, a paradigm shift in CNS tumor classification happened [6]. For the first time, tumors of the CNS were classified based on their molecular and histological signatures. Significant changes were introduced, particularly in the classification of diffuse gliomas; these gliomas were now characterized based on isocitrate dehydrogenase (IDH) 1/2 mutation and the presence of 1p/19q co-deletion. The 5th edition of the WHO classification of tumors of the CNS (WHO CNS5) from 2021 expanded on this [7]. Gliomas, glioneuronal tumors, and neuronal tumors are now grouped together, and are divided into 6 distinct families: (1) adult-type diffuse gliomas; (2) pediatric-type diffuse low-grade gliomas; (3) pediatric-type diffuse high-grade gliomas; (4) circumscribed astrocytic gliomas; (5) glioneuronal and neuronal tumors; and (6) ependymomas. Classification of high-grade gliomas is presented in Table 1.

As previously mentioned, despite multidisciplinary treatment approaches, the overall survival of GBM patients has not significantly improved over the years [3]. The current treatment includes maximal neurosurgical resection, which is followed by radiotherapy and chemotherapy [8]. Several compounds have shown promising results in vitro [9,10,11,12]. However, these results are yet to be replicated in GBM patients, and further research is needed.

Several factors have been associated with the increased or decreased risk of being diagnosed with a high-grade glioma, although controversies with studies evaluating risk and protective factors for GBM are still debated. The factors correlated with an increased or decreased risk for GBM are presented in Table 2.

### 1.2. Risk Factors

Non-modifiable risk factors. Advanced age has consistently been associated with many metabolic diseases (e.g., hypertension, diabetes) and cancers, and GBM is no exception [13]. The incidence of GBM is highest between 75 and 84 years of age [14], and as the worldwide population ages, the number of patients diagnosed with this disease is expected to rise. Genetics also plays a significant role in the pathogenesis of GBM [15], which is emphasized in the 5th edition of WHO classification of tumors of the CNS (WHO CNS5), published in 2021 [7]. Some studies reported the association of increased high-grade glioma incidence with high socioeconomic status, but these findings might be confounded by other factors, such as the race and ethnicity of patients [16]. As previously mentioned, there appears to be a relatively weak correlation between GBM risk and particular ethnic and racial groups [4]. In spite of these imbalances, molecular analyses revealed a high degree of similarity between the GBM of Caucasian and Asian patients [17]. Another non-modifiable risk factor that is associated with various neoplasms, including GBM, is the tall stature [1,18,19,20]. Although the particular mechanism is still not elucidated, it is likely mediated by insulin-like growth factor (IGF) and growth hormone (GH) pathways. Coincidentally, one of the biomarkers of GBM malignancy is insulin-like growth factor binding protein-2 (IGFBP-2), which is expressed in a majority of GBMs [21]. However, the data is inconclusive and contradictory findings about the adult stature and high-grade glioma risk have also been published [20,22].

Modifiable risk factors. One of the most consistent findings regarding modifiable risk factors was the correlation of GBM diagnosis and exposure to ionizing radiation prior in life [23]. The ionizing radiation intracellularly produces free radicals, which have a potential to induce direct genetic alterations. A comprehensive review from 2013 [24] reported a 8.1–52.3 times increased risk of subsequent CNS neoplasms in a sample of children who received radiotherapy to the cranium for pediatric cancer. It should be noted that the quality of evidence for this specific review was limited, but findings from the published literature are mostly in agreement and notable [23]. The weight of an individual, especially during early adulthood, might have a contributory role for the development of gliomas. Individuals who were obese at 18 years of age had almost four times the risk of being diagnosed with a glioma compared to individuals with normal weight [20]. In contrast, women who were underweight at the age of 21 had a decreased risk of being diagnosed with gliomas later in life [25]. This association between obesity and increased glioma risk is still being elucidated, but it is proposed that the secretory and endocrine role of adipose tissue contributes to the pathogenesis of these neoplasms [26].

With an increased interest in the study of gliomas, many risk factors have been proposed to be correlated with the increased glioma risk, with inconsistent findings. Standard diagnostic procedure after more severe head trauma includes a computed tomography (CT) scan of the head and, in some cases, tumors of the CNS are anecdotally diagnosed [27]. This warrants further research regarding the association of CNS neoplasms and head traumas earlier in life. However, these studies are often poorly controlled and, so far, have not found conclusive evidence of this correlation [1]. Prospective and well-controlled studies are needed, which should provide more insights into this topic. Although cigarette smoking has been recognized as a risk factor for numerous cancers (most notably lung cancer), none of the published studies reported an increased risk of the GBM diagnosis in patients who smoke compared to non-smokers [1]. Furthermore, according to the International Agency for Research on Cancer (IARC), exposure to certain metals is considered a risk factor for carcinogenesis. So far, the most compelling evidence for increased brain tumor risk is related to lead exposure [28], although the study by Rajaraman et al. [29] does not support this hypothesis. Possible explanation for these contrasting findings is presented by Bhatti et al. [30], who suggest that individuals with particular single-nucleotide polymorphisms (SNPs) are more vulnerable to cumulative lead exposure. Notably, these SNPs are located within genes that are important for maintaining cellular oxidative status. This suggests that, in some individuals, lead exposure might increase oxidative stress and alter energy and signaling pathways, which could induce gliomagenesis [31].

### 1.3. Protective Factors

Epidemiological studies have consistently described lower GBM prevalence in female patients compared to males [1]. This suggests that female sex hormones—estrogen in particular—might exert protective effects against the pathogenesis of gliomas. Coincidentally, women who were using estrogen replacement and oral contraceptives had a decreased risk of being diagnosed with gliomas [32]. In contrast, the same study described an increased risk of meningiomas in women utilizing hormone replacement therapy. Interestingly, patients with allergies are reported to have a decreased GBM risk [1]. Meta-analysis conducted by Linos et al. [33] described reduction in glioma risk by 40% in patients with atopic disease. Although the described reduction is striking, it might be dependent on the pathohistological type of the tumor, and further well-controlled studies are needed [34]. Furthermore, reduction in GBM risk was observed in patients who routinely took specific medications. In particular, patients who used nonsteroidal anti-inflammatory drugs (NSAIDs) had a reduced risk of developing GBM [35,36]. The assumed protective effect of NSAIDs is thought to be mediated by the inhibition of prostaglandin E_2_ (PGE_2_) synthesis, which is directly involved in the development and invasive nature of GBM [37]. However, some studies did not report a reduction of GBM risk in patients who used NSAIDs [38,39], which might be due to the complexity of the disease. Furthermore, statins have shown strong anti-GBM properties in vitro by inhibiting tumor growth through various mechanisms [40]. The only study describing the association of statin use and reduced glioma risk in patients was published by Ferris et al. [36]. In contrast, a paper by Cote et al. [41] did not report any benefits of statin use in terms of reduced glioma risk. Similarly, studies evaluating GBM risk in antihistamine users report contrasting results. Schlehofer et al. [42] and McCarthy et al. [43] reported that antihistamine users had a notable reduction of glioma risk. Surprisingly, Scheurer et al. [44] reported an increased glioma risk in patients with regular, long-term antihistamine therapy. Taken together, studies evaluating the association of specific drug and glioma risk are often inconsistent, which could be due to many variables. The explanation for these discrepancies is likely due to the molecular and histological complexity of GBM, as well as the heterogeneity of the studied sample. The complex nature of this aggressive tumor is appreciated and reflected in WHO CNS5 [7].

## 2. Metabolomics in Cancer Research

### 2.1. Mass Spectrometry

Although insight into the genetic makeup of GBM cells is indispensable in treatment of this disease, it does not provide sufficient information about the phenotype and metabolic profile of those same cells. There are several laboratory methods which are able to provide ample information about the metabolome of a tissue. One of the most widely used is mass spectrometry (MS). MS is an analytical technique that measures the mass-to-charge ratio (*m/z*) of ions and enables the determination of the precise molecular weight of a given compound, as well as biomolecules, polymers, and other particles, including products of fragmentation of investigated molecules, in order to confirm their structure [45]. The critical step in MS is the ionization of the sample molecules. After this step, molecules from the sample either divide into charged fragments or become charged without fragmentation. Due to their charge, ions can be separated based on their *m/z* ratio by accelerating and then subjecting them to an electric or magnetic field, after which they are detected, for example, by an electron multiplier. A visual representation of the ion signal as a function of the mass-to-charge ratio is known as a mass spectrum. These spectra are then utilized to specify the chemical content of a sample. There are several ionization techniques that are commonly used for chemical analysis of biological samples, such as matrix-assisted laser desorption/ionization (MALDI), surface-enhanced laser desorption/ionization (SELDI), laser desorption/ionization (LDI), and electrospray ionization (ESI) [46]. Notably, ionization techniques, which are routinely used in imaging MS (IMS), are MALDI, desorption electrospray ionization (DESI), and secondary ion mass spectrometry (SIMS) [47].

### 2.2. Matrix-Assisted Laser Desorption/Ionization Time-of-Flight Mass Spectrometry

MALDI is a soft ionization method that is similar to ESI—they both result in low fragmentation of large molecules in the gas phase. In other words, MALDI ionizes the particles itself without fragmentation. However, MALDI utilizes a laser energy absorbing matrix to produce charged particles from the sample with minimal fragmentation [48]. It is successfully applied in analyses of more fragile compounds, such as biomolecules and organic compounds. The procedure itself consists of three sequential steps. In the first step, the sample of interest is embedded in a matrix compound, after which it is applied onto a target plate. The second step consists of irradiation of the sample by a pulsed laser, which provokes ablation and desorption of the sample and matrix material. In the final step, ablated molecules are ionized, accelerated, and finally detected into a mass spectrometer. Time-of-flight mass spectrometer (TOFMS) provides a large mass range and is the most commonly used mass spectrometer with MALDI [49]. The fundamental idea behind TOFMS is that ions with distinct *m/z* are scattered in time intervals during their flights over a field-free drift route of known length. Consequently, if all ions start their flights at the same moment, or within a suitably narrow time period, lighter ions should reach the detector before heavier ions do.

**Figure 1:**
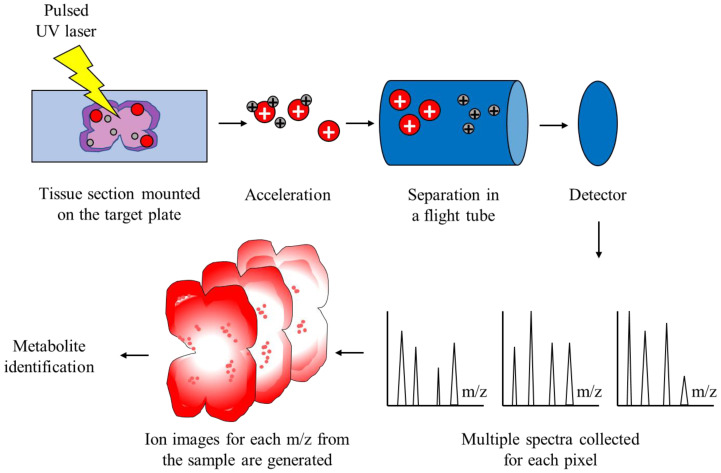
Schematic representation of MALDI-TOF IMS. The sample is embedded in a matrix solution and applied onto the target plate. Spots on the target plate are pulsed with an ultraviolet laser, which induces ablation and desorption of the molecules, which are now ionized and accelerated in an electrical field. Ionized molecules are separated in a flight tube without an electric field based on their *m/z*. The time of flight, which is correlated with the mass of particles, is captured by a detector and the final spectrum is generated for each pixel of the sample. Finally, metabolites are identified according to the generated spectra.

MALDI-TOF MS has already proven its usefulness in a variety of laboratory applications. It is suitable for the characterization of fragile proteins, which fragment when ionized by other ionization methods. MALDI-TOF enables mass determination of intact proteins with sufficient accuracy, which could then be used for sequence validation. After these proteins are digested into smaller peptides, those peptides might be analyzed with MALDI-TOF MS for primary sequence confirmation. Hence, this mode of MS is widely used in proteomics in order to analyze proteins from the mixture by a method known as peptide mass fragmentation [50]. This is possible due to impressive technical specificities of the method—it provides high resolution and sensitivity, as well as good mass accuracy. Another useful application is identification and imaging of biomolecules from thin sections of the tissue of interest, which is known as MALDI-TOF IMS, and the workflow is depicted in Figure 1 [51]. MALDI-TOF IMS combines the data generated by MALDI-TOF MS and the ability to visualize hundreds of molecules in an analyzed tissue sample, without disrupting the tissue integrity. It is important to correctly annotate the detected molecules represented by their *m*/*z* quotients. For this purpose, several automated identification pipelines have been developed, such as the METASPACE platform [52]. Furthermore, publicly available databases, such as Human Metabolome Database [53] or LipidMaps [54], can also be utilized for metabolite identification. A wide variety of molecules can be analyzed: peptides and proteins, lipids, oligonucleotides, smaller intracellular metabolites, and others. The spatial distribution of biomolecules in a sample can also be obtained, while the structural integrity of cells in a sample is preserved. Properties of MALDI-TOF MS, such as the ability to examine, relatively quickly, a large number of samples simultaneously without the need for extensive sample preparation, make it ideal for cancer research. More specifically, by analyzing samples from cancer patients, this technology enables the discovery of novel diagnostic and prognostic biomarkers and therapeutic targets for those diseases. Furthermore, by utilizing imaging alongside MALDI-TOF, researchers are able to better understand the pathophysiology of neoplasms, metabolic adaptations that occur, and molecular heterogeneity within the tumor itself.

### 2.3. MALDI-TOF MS in Cancer Research

Recent developments in analytical techniques, e.g., MALDI-TOF MS, significantly improved our understanding of cellular metabolism. These techniques, which are collectively known as metabolomics, provide valuable insights into metabolic profiles of healthy cells, as well as tumor cells [55]. Metabolomic approaches enable the detection of hundreds, and even thousands of metabolites in an analyzed sample, which could aid in developing personalized tumor therapies [56]. Application of MALDI-TOF IMS in metabolic studies of tumors advanced diagnostic and therapeutic approaches to numerous neoplasms of the gastrointestinal tract (colon, stomach, and pancreas), breast, lung, skin, thyroid, kidney, prostate, ovary, and many others, which is reviewed by Kriegsmann and colleagues [51].

Lung cancer is the most common malignancy and the leading cause of cancer-related deaths in the world [57]. It presents distinct histological subtypes; however, non-small cell lung cancer (NSCLC) is the most prevalent, accounting for more than 80% of all lung cancers [58]. The detection of specific somatic mutations in lung cancer is crucial in directing further treatment for a patient, despite the fact that there are some nuances with these neoplasms. Similar to GBM and high-grade gliomas, NSCLC are histologically heterogenous; cells presenting with a particular mutation might be a minority in the whole tumor tissue [59]. Another similarity to CNS neoplasms is the fact that obtaining tumor tissue of sufficient quality is rather complicated. Therefore, the ideal diagnostic method should be able to simultaneously screen and detect multiple mutations in a sample of limited quality. Interestingly, MALDI-TOF MS has already shown promising results. In a study by Bonaparte et al. [60], MALDI-TOF MS enabled the detection of the most prevalent mutations in NSCLC in low-quality samples. This suggests that, after proper validation, MS could be routinely used for examining lung cancer biopsies, but also for less-invasive samples, e.g., liquid biopsies. Furthermore, metabolomic information obtained by MALDI-TOF imaging MS could be used to classify NSCLC into adenocarcinoma and squamous cell carcinoma with high accuracy [61]. Curiously, this method had even higher accuracy than the current gold standard, which is immunohistochemistry. Tumor-derived extracellular vesicles (EVs), which are structures that originate from plasma membranes and facilitate communication between cells, can also be analyzed by MALDI-TOF MS [62]. Indeed, Jung et al. [63] analyzed EVs shed by NSCLC with MALDI-TOF MS. These EVs were distinguishable based on their phospholipid contents, and their phospholipidomes were predictive of treatment response. In addition, proteomic analysis of serum-derived EVs of cancer patients and healthy individuals identified seven upregulated proteins in EVs of cancer patients, one of which was protein CD5L, which might act as a potential biomarker for the early detection of this disease from serum samples [64].

Prostate cancer, right after lung cancer, is the second most frequent cancer in men [65]. The clinical suspicion of prostate cancer is based upon elevated levels of prostate-specific antigen (PSA). However, many individuals present with false-positive findings of elevated PSA, which warrants additional diagnostic procedures, namely prostate tissue biopsy, which is an invasive procedure [66]. It would be beneficial to develop a less-invasive method for the diagnosis of prostate cancer, which would inherently facilitate earlier diagnosis. Once again, MALDI-TOF MS produced promising results. Buszewska-Forajta and colleagues analyzed lipids in urine samples of patients with prostate cancer and healthy individuals [67]. By using MALDI-TOF MS, researchers were able to discriminate samples from two groups with high accuracy, ranging from 83.3% to 100%. In a similar study, Xi and colleagues also analyzed lipid contents of urine samples from patients with prostate cancer and individuals with benign prostatic hyperplasia [68]. Interestingly, they also identified two lipid types as potential biomarkers, which can be assessed non-invasively for this disease. It should be noted that prostate cancer tissue is also heterogeneously structured, which presents a problem for methods that use traditional bulk analysis. MALDI-TOF IMS could circumvent this problem by providing spatial information about metabolic peculiarities within the same tumor sample. In fact, in a study by Andersen and associates, MALDI-TOF IMS of prostate cancer emphasized the spatial differentiation of metabolic profiles within the same tumor tissue and proposed several diagnostic and prognostic biomarkers [69].

Metabolomic studies of tumor samples improved our understanding of other neoplasms as well. Breast cancer is the most common cancer in women and one of the leading causes of mortality [70]. Classification of tumors is essential in diagnosis and treatment for patients, but traditional histopathological classification is frequently imprecise due to similar histological properties of some tumors, as well as heterogeneity of cells within the tumor [71]. Determining the status of human epidermal growth factor receptor 2 (HER2) from breast cancer tissue is invaluable in providing targeted treatment [72]. In a study by Rauser et al. [73], MALDI-TOF IMS was successful in determining HER2 status of the tumor, which could, in the near future, be used for determining targeted therapeutic options. The other diagnostic issue related to newly diagnosed tumor is the correct identification of the source of the tumor; in other words, is the tumor metastatic in nature or of primary origin. Remarkably, MALDI imaging based on proteomic signatures of tumor samples successfully classified tumors as either primary or metastatic [74]. Taken together, these studies suggest that MALDI-TOF IMS has the potential to be used as a diagnostic method for the classification of different tumor types. Additional benefit of this method over conventional ones is the ability to identify previously unrecognized biomarkers [75]. Additionally, response to therapy can also be evaluated. In particular, MALDI imaging permits visualization of the spatial distribution of the anticancer drug and its metabolites in the target tissue, but also of potential toxicity in other non-targeted tissues [76]. As discussed by Lee et al. [75], standard methods used in pathology lack the ability to provide insights into heterogeneity within a tumor, while at the same time tumors that are histologically identical sometimes differ in outcomes and responses to treatment. In the near future, these invaluable complexities could very well be assessed with MALDI, as well as the response of tumor tissue, which would ultimately improve treatments.

## 3. MALDI Imaging in CNS Tumor Research

The histological environment of the GBM is complex, consisting of migrating pleomorphic tumor cells, neovascularizing tissue, local inflammatory immune cells, and necrosis [77]. The traditional histological diagnosis of GBM is based on two characteristics: neovascularization towards a common area (usually hyper-cellular zone) and necrosis, which discriminates GBM from anaplastic astrocytoma [78]. By using MALDI IMS, Ait-Belkacem et al. [79] were able to identify these two characteristics and discriminate tumor tissue and healthy surrounding tissue. Remarkably, the distinction was identified at almost the cellular level, at the spatial resolution of 30 µm. The peculiar microenvironment of GBM promotes angiogenesis, supports further expansion of the tumor, and could offer us insights into resistance to therapy [80]. Better understanding of the microenvironment could better elucidate tumor margins. Indeed, identifying tumor margins is essential in neurosurgical treatment of patients with GBM. The aggressive nature of GBM is a direct consequence of its infiltrative properties and, as of yet, these cannot be reliably evaluated with standard imaging techniques [81]. Intraoperative MS techniques, such as DESI, can advise neurosurgeons for the extent of the tumor resection, with almost real-time feedback [82]. Indeed, rapid and automated high-throughput systems, such as the one based on DESI MS, can be used to evaluate the presence of tumor cells in the studied glioma sample [83]. Interestingly, in the same study, a similar system was also utilized to determine IDH mutation status in glioma samples [83]. The aggressive nature and resistance to current therapeutics of GBM might be largely associated with GBM stem cells [84]. It is speculated that forcing the differentiation of this particular group of cells within GBM tissue could be a novel therapeutic approach for GBM treatment [85]. Theoretically, MALDI-TOF IMS might aid in this research by identifying new molecular targets of GBM stem cells.

Traditional prognostic markers of GBM, such as O(6)-methylguanine-DNA methyltransferase (MGMT) methylation, IDH mutation, epidermal growth factor receptor (EGFR) variant III (EGFRvIII), phosphatase and tensin homolog (PTEN) deletion, and vascular endothelial growth factor (VEGF) expression, improved our understanding of the disease; however, the fact of the matter is that the overall survival of patients has not significantly changed over the years [86]. An additional challenge for evaluating these markers is the need for invasive sampling. Circulating plasma is a convenient, minimally invasive sample that could theoretically provide insights into metabolic alterations correlated with GBM. In fact, Zhao et al. [87] identified 29 compounds in plasma samples that could distinguish low-grade from high-grade glioma patients. Furthermore, in the same study, IDH mutation status was correlated with just six plasma-derived metabolites. Although promising, the main limitation of plasma metabolomics is the ambiguity of identified metabolite differences. In other words, researchers cannot unequivocally conclude the source of these differences. Hence, results should be replicated and validated in other laboratories before entering clinical diagnostics.

The MALDI-based multi-omics approach consistently improves our understanding of the pathophysiology of GBM. In a recent study by Ravi et al. [88], a spatially resolved approach elucidated the bidirectional tumor-host dependence of GBM. They have concluded that transcriptional alterations are a consequence of changing local microenvironments. Furthermore, metabolic adaptation of tumor cells is a hallmark of cancer, which can also be investigated with MALDI imaging to better understand the metabolic insults occurring in tumor cells and to suggest therapeutic targets. GBM cells adapt their metabolism for increased proliferation by upregulating production of biosynthetic substrates [89]. This is supported by a recent MALDI IMS study [90] which described increased signals of glucose 6-phosphate/fructose 6-phosphate, which reinforces the Warburg effect hypothesis [91], stating that cancer cells exhibit upregulated glycolysis [92]. The same study by Dilillo et al. [90] detected increased signals of compounds involved in nucleotide metabolism, which also supports increased proliferation. MALDI-based lipidomic studies of GBM samples have both therapeutic and diagnostic importance. In fact, investigating lipidomic alterations of tumor cells can be used to evaluate the treatment effects on GBM [93]. Additionally, Maimó-Barceló and colleagues [94] proposed that, by analyzing alterations in the lipid profile of GBM and healthy brain tissue, we should be able to better understand the detrimental effects of temozolomide on healthy brains, and even reveal potential treatment options for GBM. Furthermore, assessing microvascular patterns and proteomic distribution of GBM may assist in the prediction of prognosis [95]. Even though great progress in MALDI-TOF imaging MS research of GBM has already been achieved, there is still room for future progress.

## 4. Future Perspectives of MALDI-TOF IMS in GBM Research

**Figure 2:**
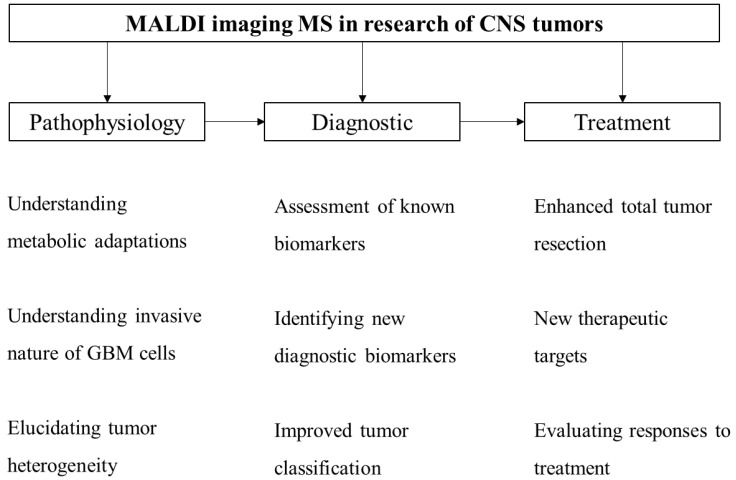
MALDI imaging MS in research of CNS tumors could improve our understanding of the pathophysiology of the disease, which could enhance diagnostic process and ultimately improve treatment options and overall survival of GBM patients.

Modern medicine is highly dependent on novel technologies [96]. As previously stated, with the wider implementation of MALDI-TOF IMS, we could improve our diagnostic and therapeutic response to GBM and, at the same time, understand the pathophysiology of the disease by identifying new biomarkers—metabolic adaptations occurring in tumor cells—and even enhance the classification of brain cancers (Figure 2). It is essential that the results obtained by MALDI-based IMS are extensively replicated and validated before introduction into everyday practice.

Despite the prior general inclination to think of a tumor as a simple collection of tumor cells, the reality is different. In most cases, neoplasms are highly complex structures which makes them hard to study with conventional laboratory methods, which are often based on bulk analysis. The preferred method should combine histopathological and metabolic analysis, and that is exactly what MALDI-TOF IMS enables. Even though the most recent classification of tumors of the CNS combines histopathology and genetics, it still does not incorporate metabolic differences between these neoplasms. Identifying distinct metabolic insults in tumors of the CNS could be of great interest and could provide us with new therapeutic targets for which specific drugs may be developed. A study by Petre and colleagues [97] provides an example of how proteomic profiling can help in distinguishing malignant cell lines of GBM. The ultimate and desired result would be better outcomes for patients with high-grade gliomas, and GBM in particular.

Although MALDI-TOF IMS already achieved promising results in pre-clinical settings, implementation of the method in clinical settings is still limited. The main reason for relatively slow implementation in everyday practice is high instrument and maintenance cost [75]. In addition, operating the machinery is technically difficult and demands a highly skilled workforce. The matrix used in MALDI might interfere with signals of compounds with low molecular weight, such as drugs and metabolites [98]. In most cases, the majority of processing is performed manually, which prolongs the process and introduces inherent variance related to manual processing. There are reports of analysis-related variability even in a single laboratory. Incorporating automation is needed to increase efficiency and reduce variability. For this reason, findings from pre-clinical studies should be extensively replicated and validated before implementation in a clinical setting.

The data generated by MALDI-TOF MS is extensive and should be appropriately investigated. However, the analysis is challenging due to limited computational methods and databases, which calls for a high-throughput computational pipeline [99]. Machine learning and deep learning implementation in the pipeline significantly improves the efficiency and reduces the need for manual work in the process [100]. It should also be noted that MALDI imaging is mostly performed on thin tissue sections, which are considered two-dimensional (2D). This 2D approach might not be adequate for complex and heterogeneous structures, such as tumors, which should be studied in three-dimensional (3D) space. This challenge has already been addressed by researchers who developed 3D imaging techniques and successfully used it in cancer research [101]. Imaging performance employed by MALDI-TOF IMS also has some limitations [75]. This pitfall could be addressed by a combined approach—merging MALDI imaging with other techniques—which improves the quality of images and provides supplementary insights about the molecular characteristics of the studied sample [102].

## 5. Conclusions

In this review, we have summarized the workflow of MALDI IMS and emphasized the potential of this technology in GBM research.

GBM is a devastating disease for which modern medicine has yet to discover effective treatment. Metabolomic techniques have significantly improved our understanding, diagnostic and therapeutic approaches to various malignancies. Great progress has been made in metabolomic studies of GBM. MALDI IMS could be an invaluable tool in understanding the pathophysiology of this aggressive brain tumor, which might further improve diagnostic and treatment modalities.

## Figures and Tables

**Table 1:** Adult- and pediatric-type high-grade gliomas according to the 2021 WHO classification of Tumors of the Central Nervous System.

**Adult-type diffuse gliomas**
Astrocytoma, IDH-mutant
Oligodendroglioma, IDH-mutant and 1p/19q-codeleted
Glioblastoma, IDH-wildtype
**Pediatric-type diffuse high-grade gliomas**
Diffuse midline glioma, H3 K27-altered
Diffuse hemispheric glioma, H3 G34-mutant
Diffuse pediatric-type high-grade glioma, H3-wildtype and IDH-wildtype
Infant-type hemispheric glioma

**Table 2:** Risk and protective factors for glioblastoma.

Non-Modifiable Risk Factors	Modifiable Risk Factors	Protective Factors
Age	Exposure to ionizing radiation	Female sex hormones
High socioeconomic status	Weight	History of allergies
Ethnicity and race	Head trauma	Medications: NSAIDs Statins Antihistamines
Tall stature	Exposure to metals (lead)	

NSAIDs: nonsteroidal anti-inflammatory drugs.
